# A sustained release of BMP2 in urine-derived stem cells enhances the osteogenic differentiation and the potential of bone regeneration

**DOI:** 10.1093/rb/rbac015

**Published:** 2022-04-25

**Authors:** Shuang Wu, Zhao Chen, Xi Yu, Xin Duan, Jialei Chen, Guoming Liu, Min Gong, Fei Xing, Jiachen Sun, Shishu Huang, Zhou Xiang

**Affiliations:** 1 Department of Orthopedics, Orthopedic Research Institute, West China Hospital, Sichuan University, Chengdu 610000, China; 2 Rehabilitation Medicine Center, West China Hospital, Sichuan University, Chengdu 610000, China; 3 Department of Orthopedics, Affiliated Hospital of Qingdao University, Qingdao 266000, China; 4 Department of Orthopedics, Hospital of Chengdu University of Traditional Chinese Medicine, Chengdu 610000, China

**Keywords:** urine-derived stem cells, BMP2, chitosan microspheres, bone tissue engineering

## Abstract

Cell-based tissue engineering is one of the optimistic approaches to replace current treatments for bone defects. Urine-derived stem cells (USCs) are obtained non-invasively and become one of the promising seed cells for bone regeneration. An injectable BMP2-releasing chitosan microspheres/type I collagen hydrogel (BMP2-CSM/Col I hydrogel) was fabricated. USCs proliferated in a time-dependent fashion, spread with good extension and interconnected with each other in different hydrogels both for 2D and 3D models. BMP2 was released in a sustained mode for more than 28 days. Sustained-released BMP2 increased the ALP activities and mineral depositions of USCs in 2D culture, and enhanced the expression of osteogenic genes and proteins in 3D culture. *In vivo*, the mixture of USCs and BMP2-CSM/Col I hydrogels effectively enhanced bone regeneration, and the ratio of new bone volume to total bone volume was 38% after 8 weeks of implantation. Our results suggested that BMP2-CSM/Col I hydrogels promoted osteogenic differentiation of USCs in 2D and 3D culture *in vitro* and USCs provided a promising cell source for bone tissue engineering *in vivo*. As such, USCs-seeded hydrogel scaffolds are regarded as an alternative approach in the repair of bone defects.

## Graphical Abstract

**Graphical Abstract:**
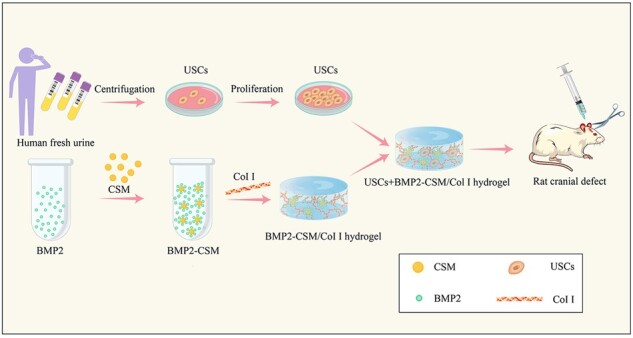

## Introduction

Large bone defects associated with trauma, tumor and infection frequently require surgical intervention [1, 2]. Currently, the transplantation of autologous bone is still the gold standard treatment for bone defects due to its osteoconductive, osteoinductive and osteogenic properties, which are necessary for bone regeneration [3]. However, the disadvantages are inevitable, including the limited tissue availability, transmission of diseases and additional surgical injuries [4]. Therefore, tissue-engineered bone grafts have been designed and developed to enhance bone regeneration [5].

Bone tissue engineering aims to induce new bone regeneration by the combination of biodegradable scaffolds, living cells and signaling molecules [6]. Stem cell-based therapy has been considered as a promising strategy for bone repair with the characteristics of self-renewal and multi-directional differentiation potential [7]. Additionally, stem cells promoted the repair of injured tissues by the secretion of growth factors, micro-environmental modification, resident cell recruitments, immunoregulation and vascularization. Many kinds of stem cells have been considered as the ideal therapeutic sources, including bone marrow-derived mesenchymal stem cells (MSCs), umbilical cord MSCs, adipose-derived stem cells, *etc*. However, insufficient sources, inconvenient access, invasive procedures and safety concerns have become the main obstacles in their application [8].

Interestingly, urine-derived stem cells (USCs) are isolated from fresh urine by a non-invasive, reliable, simple, cost-effective and safe method [9]. It has been reported USCs share similar biological properties with MSCs and possess the capacities of robust proliferation and multi-potential differentiation [10]. USCs have been applied in the regeneration of urological system, musculoskeletal system, brain, skin, hepatic tissue and so on, and the implantation therapy of autologous USCs avoids immunological rejection [11]. One of our previous studies showed the chitosan-optimized biphasic calcium-phosphate scaffold loaded with USCs could promote the repair of large bone defects effectively [12]. Therefore, these suggest USCs have great potential for bone defect repair.

It is known that micro-environments have been involved in cellular growth, proliferation, migration and other cellular behaviors. The scaffolds in micro-environments are highlighted due to their bioactive characteristics *in vitro* and *in vivo* [13]. Type I collagen (Col I) is an excellent alternative of biomaterials in bone tissue engineering for its good biocompatibility and osteoconductivity, and it has been reported that cell-seeded Col I hydrogels could significantly promote bone healing [14]. Bone morphogenetic protein 2 (BMP2) has been reported to enhance the proliferation, recruitment and osteogenic differentiation of MSCs [15]. Conversely, the excessive concentration of BMP2 after a burst release caused ectopic bone formation or inflammation [16, 17]. Chitosan has been widely processed into chitosan microspheres (CSMs) for the sustained release of BMP2 [18, 19]. However, the cellular behaviors and osteogenic differentiation of USCs stimulated by BMP2 in 2D and 3D culture remained elusive.

In this study, we fabricated an injectable BMP2-releasing chitosan microspheres/type I collagen hydrogel (BMP2-CSM/Col I hydrogel), and investigated its biocompatibility and osteogenic effects on USCs in 2D and 3D culture. A sustained release of BMP2 promoted osteogenic differentiation of USCs through the BMP2/p-Smad signaling pathway *in vitro*. The combination of USCs and BMP2-CSM/Col I hydrogels promoted bone formation efficiently compared with Col I, BMP2-CSM/Col I and USCs + Col I hydrogels *in vivo*.

## Materials and methods

### Cell isolation and culture

The study was approved by the Ethics Committee of West China Hospital, Sichuan University (2020333A). Sterile urine samples were collected from three healthy adult male donors (age: 23–27 years old). Primary USCs were obtained and cultured using the methods described previously [20, 21]. Briefly, each urine sample (about 200 ml) was centrifuged at 491 × *g* for 10 min, and the supernatant was discarded. Cells were then resuspended in 20 ml of phosphate-buffered saline (PBS), washed, and centrifuged at 491 × *g* for 5 min. The supernatant was discarded, and cells were washed using PBS again. Then, cells were directly seeded onto 24-well plates with improved medium comprised of 50% K-SFM (Gibco, Thermo Fisher Scientific, USA), 33.75% DMEM-HG (Gibco, Thermo Fisher Scientific, USA), 11.25% Ham’s F12 (Hyclone, GE Healthcare Bio-Sciences, USA), 5% fetal calf serum (FBS; Gibco, USA), 1% penicillin-streptomycin (Gibco, USA) and some supplements. Cells were cultured in a 5% CO_2_ incubator at 37°C. Single-cell clones appeared on the plates after culture for 3–5 days. Cells were digested, collected and passaged by using trypsin after reaching subconfluency. Passage 3-4 (P3-4) USCs were used for the following experiments.

### Characterizations of USCs

#### Cell proliferation

USCs were seeded onto 96-well plates at a density of 2500 cells/well and assessed for 10 days using Cell Counting Kit-8 (CCK-8, Dojindo Molecular Technologies, Japan). Briefly, cells were incubated with 100 µl of FBS-free medium containing 10 µl of CCK-8 solution at 37°C for 3 h. After incubation, the supernatant was transferred to a new 96-well plate, and the optical density (OD) values were measured at 450 nm using a spectrophotometer.

#### Flow cytometry analysis

1 × 10^6^ USCs were resuspended in 100 µl PBS, and then incubated with the following fluorochrome-conjugated monoclonal antibodies (1:50; BD Pharmingen, USA) including CD34 (No. 561 440), CD14 (No. 557 831), CD31 (No. 563 653), CD45 (No. 557 833), HLA-DR (No. 560 743), CD105 (No. 563 920), CD90 (No. 561 970), CD29 (No. 561 794), CD44 (No. 560 977) and CD73 (No. 561 014) at 4°C for about 30 min in the dark. Cells were centrifuged, and washed with PBS after discarding the supernatant. Finally, USCs were resuspended in 300 µl PBS, detected using Flow Cell Sorter (BD, USA), and then analyzed with FlowJo_V10 software.

#### Multi-lineage differentiation

Cells were seeded onto 24-well plates at a density of 2 × 10^4^ cells/well. For the test group, cells were cultured with osteogenic, chondrogenic and adipogenic differentiation media (Cyagen, China) after reaching 60–70% confluence, respectively. For the control group, cells were cultured with USCs media. After induction for 21 days, cells were stained with Alizarin red S (ARS), Toluidine blue and Oil Red O solution (Cyagen, China) to evaluate the capability of osteogenic, chondrogenic and adipogenic differentiation, respectively.

### Preparation of CSMs

CSMs were prepared using water-in-oil emulsification and chemical cross-linking techniques [18, 22]. Briefly, 0.625 g chitosan was dissolved in 25 ml 2% (v/v) acetic acid aqueous solution. Chitosan solution was added dropwise into 125 ml liquid paraffin containing 1% (v/v) Tween80 and 3% (v/v) Span80, and then fully mixed using mechanical stirring at 800 rpm for 2 h. Subsequently, 25% (v/v) glutaraldehyde (GA) was added dropwise to the homogenous emulsion three times at the interval of 15 min (the volume of GA: 1.6, 1.6 and 3.2 ml, respectively). Microspheres were collected by centrifugation at 1372 × *g* for 5 min, and then washed with petroleum ether, methanol, isopropyl alcohol, ethanol and distilled water, respectively. Finally, microspheres were lyophilized and sterilized by ethylene oxide gas for further research.

### Preparation of BMP2-CSM/Col I hydrogels

Lyophilized CSM were loaded with BMP2 (Sino Biologial, China) via diffusional transferring and physical adsorption. Briefly, 2 mg CSM were immersed in 50 µl BMP2 solution (20 μg/ml), and then incubated overnight at 4°C. BMP2-CSM solution was collected for following experiments *in vitro* and *in vivo*.

Rat tail Col I solution (800 µl 5 mg/ml ) (Solarbio, China) was mixed with 90 µl 10 × PBS, and then neutralized with 12 µl 0.1 M NaOH under the instruction. Next, 100 µl 1 × DMEM with or without cells was added and mixed. Finally, the neutral Col I solution was kept at 37°C for about 5 min to form a 4 mg/ml Col I hydrogel. Similarly, 1 × DMEM (with or without cells) with CSM or BMP2-CSM was added into the Col I solution to fabricate CSM/Col I hydrogels or BMP2-CSM/Col I hydrogels.

### Characterizations of microspheres

Freeze-dried microspheres were spray-coated with gold, and observed under scanning electron microscopy (SEM; Zeiss, Germany). Size distribution was analyzed using a laser particle analyzer (Sympatec, Germany). To perform the swelling study of CSM, microspheres were immersed and mixed in excess deionized water at 37°C for 24 h. The dry and swollen microspheres were weighed respectively, and then swelling ratios were calculated. Swelling ratio = (weight of microspheres at 24 h −weight of microspheres at 0 h)/weight of microspheres at 0 h × 100%.

### Characterizations of BMP2-CSM/Col I hydrogels

Inverting test was performed to measure the time of gel formation. Col I, CSM/Col I and BMP2-CSM/Col I gel solution was pipetted into EP tubes respectively, and then incubated at 37°C. Hydrogels were fabricated successfully if there was no visual flow after inverting the tubes, and the time of incubation was recorded. The morphology of freeze-dried hydrogel scaffolds sputter-coated with gold was observed using SEM. To evaluate the release profile of BMP2 *in vitro*, 200 µl of BMP2-CSM/Col I hydrogel was immersed in 1 ml PBS, and then placed in an incubator shaker at 37°C. The supernatants were collected at each predetermined time point (1, 3, 5, 7, 14, 21 and 28 days), and replaced by an equal amount of fresh PBS. The concentration of BMP2 was measured by a BMP2 ELISA kit (Sino Biologial, China) according to the instruction.

### Biocompatibility of BMP2-CSM/Col I hydrogels *in vitro*

#### Cell proliferation

In 2D culture, 2800 cells/well were seeded into a 96-well plate (Corning, USA) coated with different hydrogels (Col I, CSM/Col I or BMP2-CSM/Col I hydrogel). In 3D culture, Col I, CSM/Col I and BMP2-CSM/Col I hydrogels with 1 × 10^6^/ml cells were added into a 96-well plate (Corning, USA) respectively, and then placed in a cell incubator. Culture medium was added after gel formation and changed every day. Cell proliferation was evaluated using CCK-8 at Days 1, 4, 7 and 10.

#### Live/dead staining

2 × 10^4^/well cells were seeded into a 24-well plate (Cellvis, USA) coated with Col I, CSM/Col I and BMP2-CSM/Col I hydrogels in the 2D model, and Col I, CSM/Col I and BMP2-CSM/Col I hydrogels with 1 × 10^6^/ml cells were added into a 24-well plate (Cellvis, USA), respectively, in the 3D model. All samples were stained with the Calcein-AM/PI Double Staining Kit (Dojindo Molecular Technologies, Japan) according to the operation manual at Days 1 and 4, and then visualized under a laser scanning confocal microscope (LSCM; Nikon, Japan).

#### Cell adhesion

2 × 10^4^/well cells were seeded into a 24-well plate (Cellvis, USA) coated as above-mentioned in the 2D model, and different hydrogels embedded with 1 × 10^6^/ml cells were added into a 24-well plate (Cellvis, USA) in the 3D model. Fibrous actin (F-actin) cytoskeletons of all samples were stained with 100 nM TRITC Phalloidin solution (No. 40734ES75, Yeason, China), and nuclei were further visualized using DAPI solution (No. C1005, Beyotime Biotech, China) at Days 1 and 4.

Similarly, circular glass culture plates were coated with different hydrogels in 24-well plates, and then 2 × 10^4^/well cells were seeded. At Days 1 and 4, the samples were harvested and fixed by 4% paraformaldehyde, dehydrated through a series of alcohol concentrations (10%, 20%, 30%, 50%, 70%, 85%, 90% and 100% twice), dried with CO_2_ critical-point drying, and then spray-coated with gold. Samples were observed under SEM.

#### Cell migration

The migration of USCs was evaluated using 6-well 0.4 μm transwell inserts (Corning, USA). 2 × 10^5^/well cells were seeded into the lower wells, and then cells were serum starved overnight before the scratch after reaching 95–100% confluence. Next, the cell monolayer was scraped with a 10 µl pipette tip. Col I, CSM/Col I and BMP2-CSM/Col I hydrogels were added into the upper inserts, respectively, and co-cultured with USCs using serum-free medium. The size of scratch was monitored by an inverted phase contrast microscope (Nikon, Japan) at 0, 8 and 18 h, and analyzed using ImageJ software (Version 1.52p, National Institutes of Health, USA). Remaining area ratio = remaining scratch size at 8 or 18 h/scratch size at 0 h × 100%.

### Osteogenic differentiation of USCs in 2D culture

2 × 10^4^/well cells were seeded in the lower wells of a 24-well plate. After reaching 60–70% confluence, USCs were induced using osteogenic medium (Cyagen, China). Col I, CSM/Col I and BMP2-CSM/Col I hydrogels were added into the upper inserts, respectively. At Days 4, 7, 14 and 21, cells were stained with a BCIP/NBT Alkaline Phosphatase Color Development Kit (Beyotime, China), and osteogenic USCs were stained with blue. The level of alkaline phosphatase (ALP) was quantified using an ALP assay kit (Nanjing Jiancheng Bioengineering Institute, China) according to the user manual.

Calcium deposits were visualized using ARS staining (Cyagen, China) at Days 7, 14 and 21. To quantify the calcium content, orange-red deposition was dissolved by 10% (w/v) cetylpyridine (TCI, China), and OD values were measured at 540 nm.

### Osteogenic differentiation of USCs in 3D culture

#### Real-time PCR

USCs (1 × 10^6^/ ml) were embedded in Col I, CSM/Col I and BMP2-CSM/Col I hydrogels respectively, and then induced using osteogenic medium after gelling. After 4, 7, 14 and 21 days of osteogenic induction, total RNA was isolated using the Total RNA isolation kit (Foregene, China), and reverse transcription was performed using the PrimeScript™ RT reagent Kit with gDNA Eraser (Takara, Japan). The expression levels of osteogenic genes including collagen I (COL1A1), ALP and RUNX family transcription factor 2 (RUNX2) were measured by real-time reverse transcription polymerase chain reaction (RT-PCR) using the TB Green™ Premix Ex Taq™ II kit (Takara, Japan). Table 1 shows the primer sequences. Glyceraldehyde-3-phosphate-dehydrogenase (GAPDH) was used as an internal control.

**Table 1.** Primer sequences used in RT-PCR

Gene	Primer sequence (5′–3′)
COL1A1	Forward GCCCAGAAGAACTGGTACATCAG
	Reverse CGCCATACTCGAACTGGAATC
ALP	Forward ACCACCACGAGAGTGAACCA
	Reverse CGTTGTCTGAGTACCAGTCCC
RUNX2	Forward CCAACCCACGAATGCACTATC
	Reverse TAGTGAGTGGTGGCGGACATAC
GAPDH	Forward ACAACTTTGGTATCGTGGAAGG
	Reverse GCCATCACGCCACAGTTTC

The sustained release of BMP2 increased the ALP activity and mineral deposition of urine-derived stem cells (USCs) in 2D culture, and enhanced the expression of osteogenic genes (COL1A1, ALP and RUNX2) and proteins (COL1A1, ALP and p-Smad 1/5/9) of USCs in 3D culture. *In vivo,* the mixture of USCs and BMP2-CSM/Col I hydrogels effectively enhanced bone regeneration in rat cranial defects.

#### Western blotting analysis

Similarly, total protein was extracted from 3D cultured cells using the method as previously reported [23]. The protein concentration of each sample was calculated using a BCA Protein Assay Kit (Epizyme Biotech, China). Proteins were separated by SDS-PAGE gels (Epizyme Biotech, China), and then transferred to PVDF membranes (Millipore, USA). Membranes were blocked using 5% non-fat dry milk, and then incubated with primary anbibodies overnight at 4°C, including anti-COL1A1 (1:1000; No. 66761-1-Ig, Proteintech, China), anti-ALP (1:5000; No. 381 009, ZENBIO, China), anti-phospho-Smad (p-Smad) 1/5/9 (1:500; No. 530 850, ZENBIO, China), anti-Smad 1/5/9 (1:500; No. 252 529, ZENBIO, China) and GAPDH (1:5000; No. ET1601-4, HUABIO, China). After incubating with horseradish peroxidase (HRP)-conjugated goat anti-mouse IgG (1:10 000; No. ZB-2305, Zhongshan Golden Bridge, China) or HRP-conjugated goat anti-rabbit IgG (1:10 000; No. 511 203, ZENBIO, China) at room temperature for 1 h, membranes were coated with chemiluminescent substrate solution (No. BMU102-CN, Abbkine, China) and visualized using a chemiluminescence imaging system (ChemiDoc™ MP, Bio-Rad, USA). The quantification of proteins was performed using ImageJ software (NIH, USA).

### *In vitro* antibacterial activity assays

*Staphylococcus aureus* (ATCC25923; Solarbio, China) and *Escherichia coli* (ATCC25922; Solarbio, China) were cultured in Luria Broth aerobically at 37°C. Bacterial suspensions were diluted to 1 × 10^6^ colonies forming units/ml.

Diluted bacterial suspensions (1 ml) were treated with Col I, CSM/Col I and BMP2-CSM/Col I hydrogels (300 µl), respectively, in 24-well plates. After 12 h culture, bacterial suspensions were collected, subsequently transferred to a 96-well plate. The bacterial density was measured at 600 nm using a spectrophotometer [24].

### Animal experiments

Male Sprague-Dawley (SD) rats (10 weeks old; Chengdu Dossy Experimental Animal Co., Ltd, China) were anesthetized via inhalation anesthesia with isoflurane. A 2.0 cm sagittal incision was subsequently made. Bilateral critical cranial defects (d: 5.0 mm) were drilled using a dental drill. Then, Col I or BMP2-CSM/Col I hydrogels embedded with or without 1 × 10^6^/ml USCs (Col I, BMP2-CSM/Col I, USCs + Col I and USCs + BMP2-CSM/Col I) were implanted, followed by layered closures with 5-0 absorbable sutures. Animals were sacrificed at 8 weeks after implantation, and cranial bones were harvested.

### Micro-CT

To assess bone regeneration, cranial samples of 8 weeks were scanned using the Quantum GX micro-computed tomography (micro-CT) Imaging System (PerkinElmer, USA), and 3D reconstruction was performed using Analyze 12.0 software (PerkinElmer, USA). The amount of bone defect repair was determined by the percentage of new bone volume over total volume (BV/TV).

### Histological analysis

Briefly, the samples were fixed in 4% (w/v) paraformaldehyde for 24 h, and then decalcified using EDTA decalcifying solution (Solarbio, China) for 4 weeks. The samples were washed with PBS several times, dehydrated through graded alcohol, and then embedded in paraffin wax. Next, samples were cut into 5-μm-thick sections at the center of embedded samples. Hematoxylin-eosin (HE) staining and immunohistochemical staining with primary antibodies (Abcam, USA) including anti-COL1A1 (No. ab270993), anti-osteocalcin (OCN; No. ab13420) and anti-CD31 (No. ab281583) were performed.

To trace the fate of USCs in the bone defects, we performed immunofluorescent staining using DAPI and anti-NuMA antibodies specific for human nuclei. The sections were washed, and blocked with goat serum for 20 min at 37°C. Next, the sections were incubated with anti-NuMA antibodies (1:500; No. ab109262, Abcam, USA) at 4°C overnight, followed by incubation with Rhodamine (TRITC)-conjugated Goat Anti-Rabbit IgG (H + L) (1:100; Proteintech, China) at 37°C for 1 h. Finally, cell nuclei were stained with DAPI solution (No. C1005, Beyotime Biotech, China) for 5 min. Samples were observed using a fluorescence microscope (Ti2, Nikon, Japan).

### Statistical analysis

All experiments were performed in triplicate. Statistical analyses were performed using SPSS 21.0 software (IBM Corp., USA). All data were analyzed by one-way analysis of variance, followed by subgroup analysis with Turkey HSD test. *P *<* *0.05 was regarded as statistical significance.

## Results

### Characterizations of USCs

**Figure 1.**
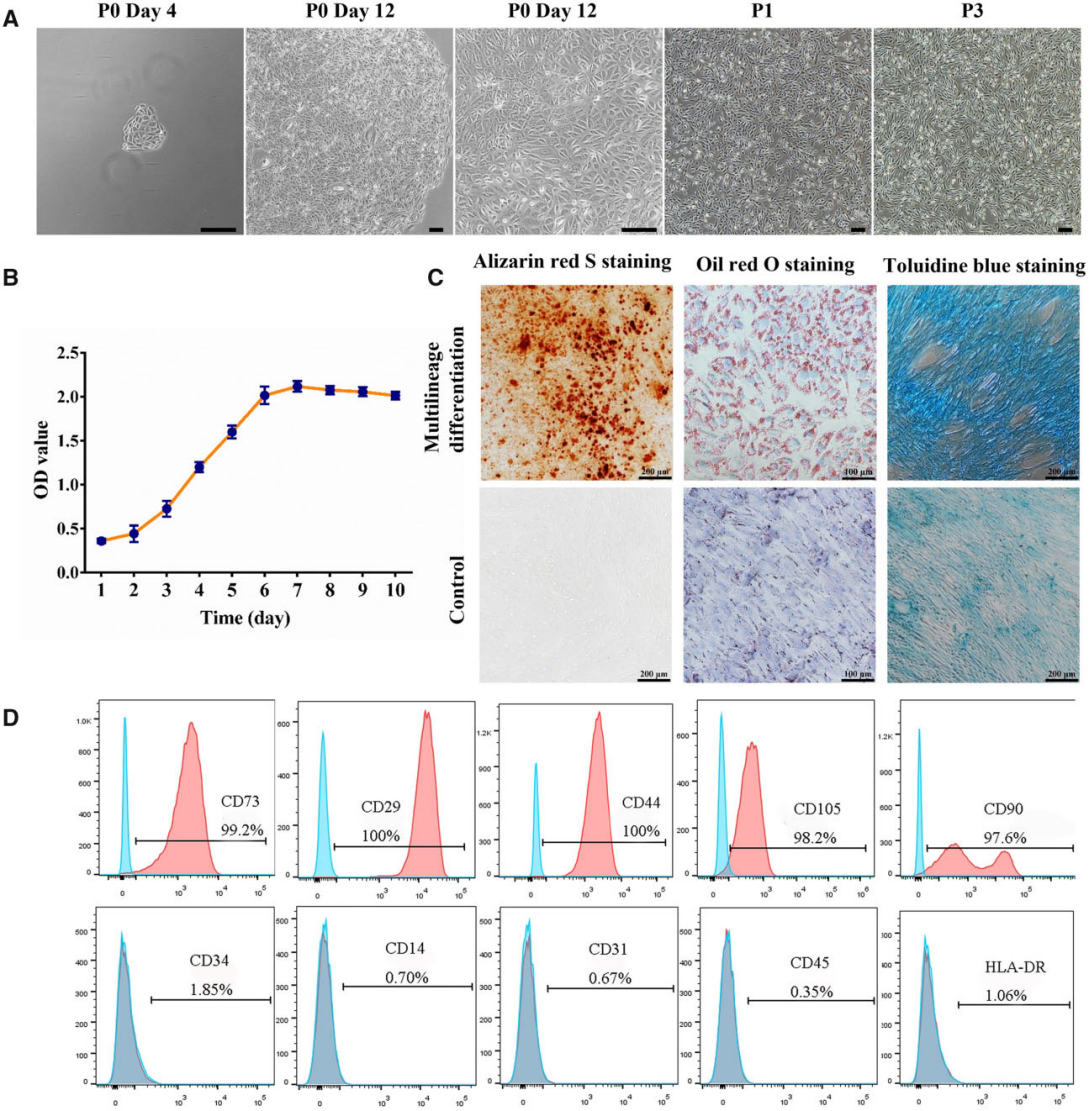
The characterizations of USCs. (**A**) The morphology of USCs. Scale bar = 200 μm. (**B**) The growth curve of P3 USCs. (**C**) The osteogenic, adipogenic and chondrogenic differentiation of USCs. (**D**) The cell surface markers of USCs. Positive markers: CD73, CD29, CD44, CD105 and CD90; negative markers: CD34, CD14, CD31, CD45 and HLA-DR

USCs were isolated from urine and plated in culture plates as described previously. Several small cell colonies were firstly observed at 3–5 days after isolation, and then grew into 90–100% confluence in the next 5–8 days (Fig. 1A). Cell proliferation was evaluated using CCK-8 solution, and the results showed spindle-shaped or rice-shaped USCs proliferated rapidly with a S-shaped growth pattern *in vitro* (Fig. 1A and B). Compared with the control group, USCs were induced to differentiate to osteogenic, adipogenic and chondrogenic lineages (Fig. 1C). Flow cytometry revealed that USCs expressed MSCs-like surface markers. USCs were positive for CD44, CD73, CD105, CD90 and CD29, and negative for CD34, CD14, CD31, CD45 and HLA-DR (Fig. 1D).

### Characterizations of microspheres

**Figure 2.**
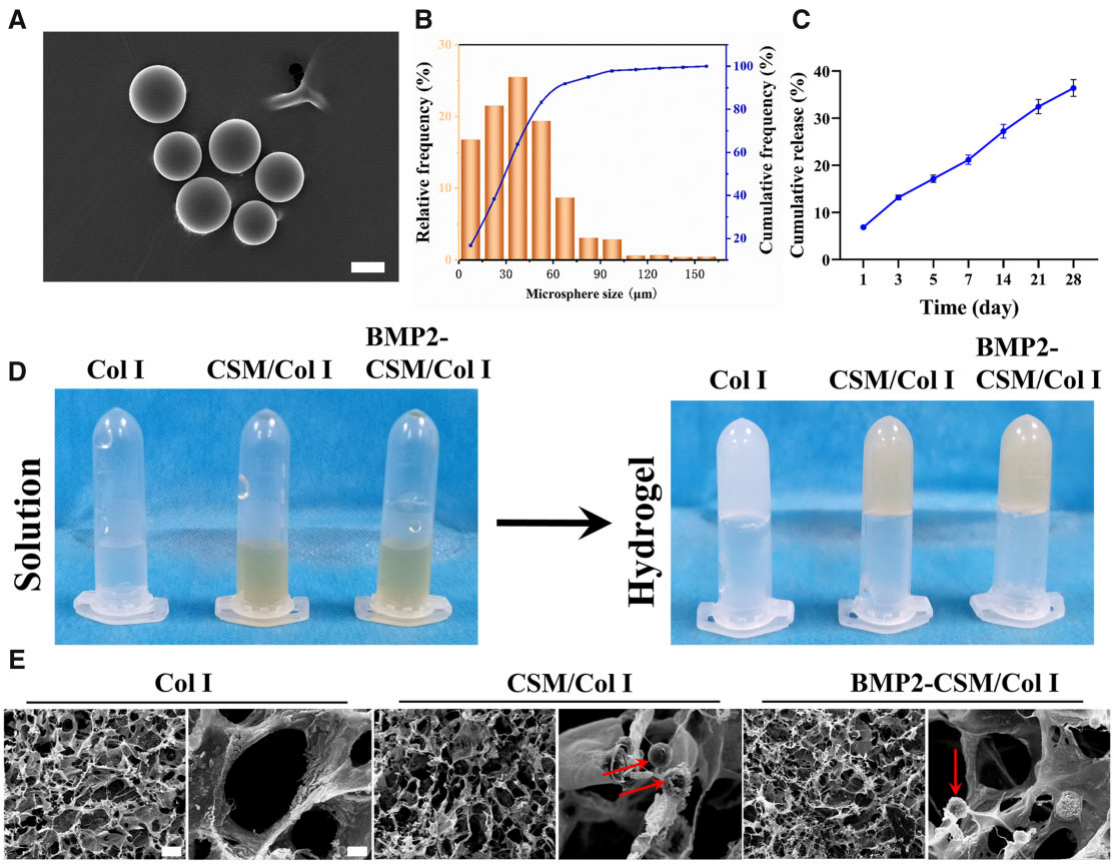
Characterizations of CSM and different hydrogels. (**A**) The morphology of CSM. Scale bar = 30 μm. (**B**) The particle size distribution of CSM. (**C**) The cumulative release of BMP2 from BMP2-CSM/Col I hydrogels. (**D**) Inverting study showed hydrogels were fabricated successfully. (**E**) The morphology of freeze-dried hydrogels. Arrows represent microspheres. Scale bar in the left column of images of each hydrogel is 100 μm. Scale bar in the right column of images of each hydrogel is 10 μm

In order to incorporate BMP2, we prepared CSMs as described previously, and determined key physical properties of the spheres. The morphology of microspheres was observed by SEM. CSM were regular spheres without cracks or adhesions (Fig. 2A). The average diameter of microspheres was 39.2 μm (Fig. 2B).

Swelling property is one of the important characteristics of CSM, which can be influenced by molecular weight, degree of deacetylation, hydrophility and cross-linking degree [18]. Swelling study showed the swelling ratio of CSM was 323.3 ± 8.2%, which was consistent with the previous study [18].

### Characterizations of BMP2-CSM/Col I hydrogels

To understand the release profile of BMP2, the release kinetics of BMP2 were studied *in vitro*. BMP2 could be released slowly from BMP2-CSM/Col I hydrogels, and no obvious burst or inactivation was observed. The cumulative release of BMP2 was 36.4 ± 1.6% at Day 28 (Fig. 2C).

According to the results of inverting test (Fig. 2D), the gelation time of Col I, CSM/Col I and BMP2-CSM/Col I hydrogels was 5.2 ± 0.6 min, 5.2 ± 0.2 min and 5.8 ± 0.2 min, respectively, and no statistical difference was found between groups.

The morphology of different hydrogels was observed under SEM (Fig. 2E). Each freeze-dried hydrogel showed porous structure and interconnected pores. Microspheres were embedded in CSM/Col I and BMP2-CSM/Col I hydrogels, and the basic structure of hydrogels was not significantly affected.

### Cytocompatibility in 2D and 3D culture

**Figure 3.**
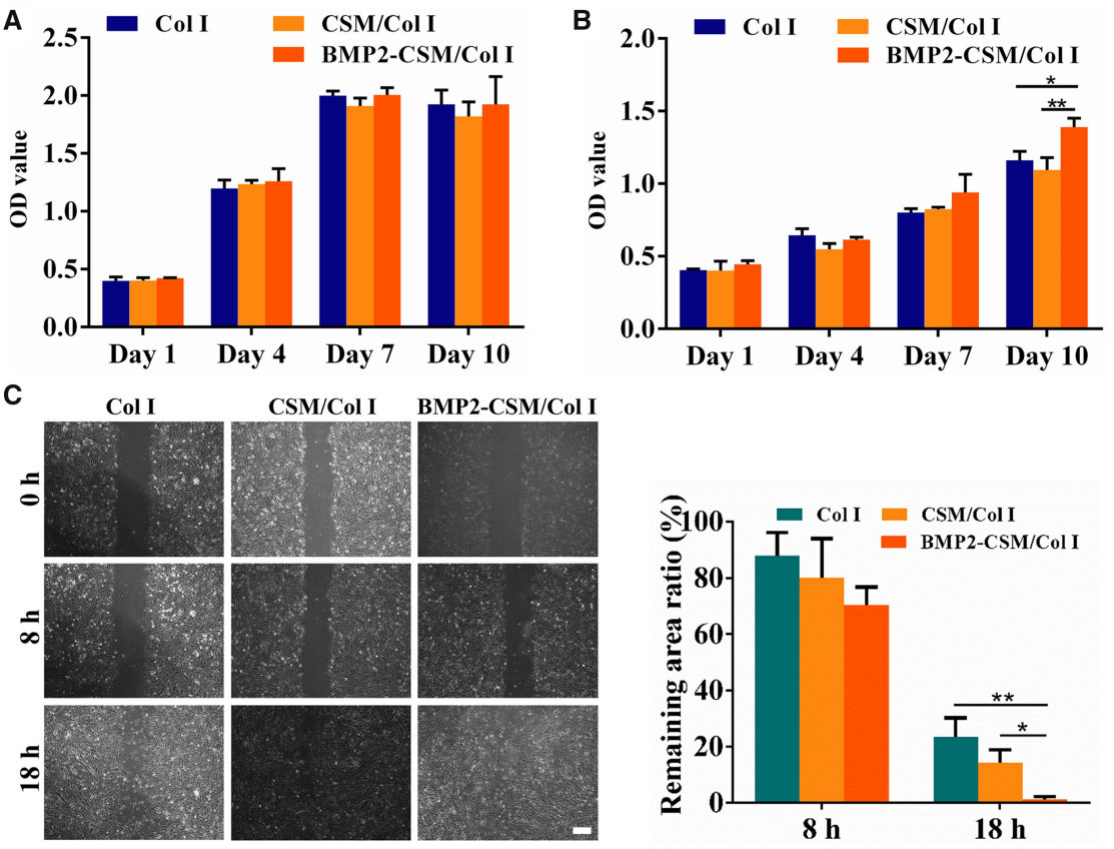
(**A**) Cell proliferation using a CCK-8 kit in 2D culture. (**B**) Cell proliferation using a CCK-8 kit in 3D culture. (**C**) Scratch wound healing assay and quantification. Scale bar = 200 μm. *P <* *0.05, **P <* *0.01

USCs were seeded on the surface of different hydrogels in 2D culture or embedded in hydrogels in 3D culture. Cell proliferation was determined by CCK-8 at predetermined intervals. The number of USCs increased both in 2D and 3D modes in a time-dependent pattern. For the 2D mode, the proliferation rate reached the peak at Day 7, and slightly decreased at Day 10. No significant difference of cell numbers was detected between groups (Fig. 3A). In 3D culture, there was no statistically significant difference between groups at Day 1, 4 and 7. However, the proliferation rate of the BMP2-CSM/Col I hydrogel group was significantly higher than other two groups at Day 10 (Fig. 3B).

Scratch wound healing assay was performed *in vitro* to evaluate the migration of USCs (Fig. 3C). Many cells migrated to the scratch area at 8 h, but no significant difference of gap closure was found between groups. At 18 h, the scratch size of each group further decreased, and the gap closure rate of the BMP2-CSM/Col I hydrogel group was statistically higher than other two groups (*P *<* *0.05).

**Figure 4.**
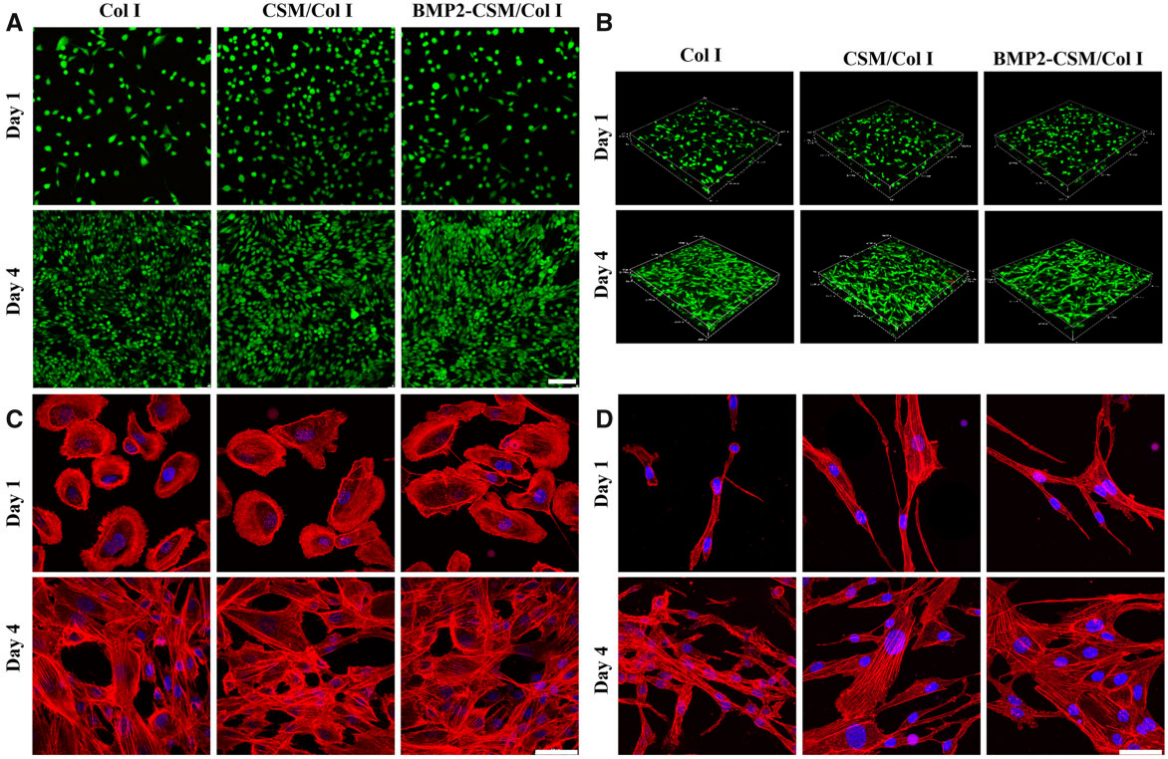
(**A**) Live/dead staining in 2D culture. Scale bar = 100 μm. (**B**) Live/dead staining in 3D culture. (**C**) Cytoskeleton staining in 2D culture. Scale bar = 50 μm. (**D**) Cytoskeleton staining in 3D culture. Scale bar = 50 μm

Cell viability was evaluated using Live/Dead staining in 2D (Fig. 4A) and 3D (Fig. 4B) culture. USCs survived and proliferated on the surface and in the interior of hydrogels, and almost no dead cell was noted in each group, which means good viability of different hydrogels.

**Figure 5.**
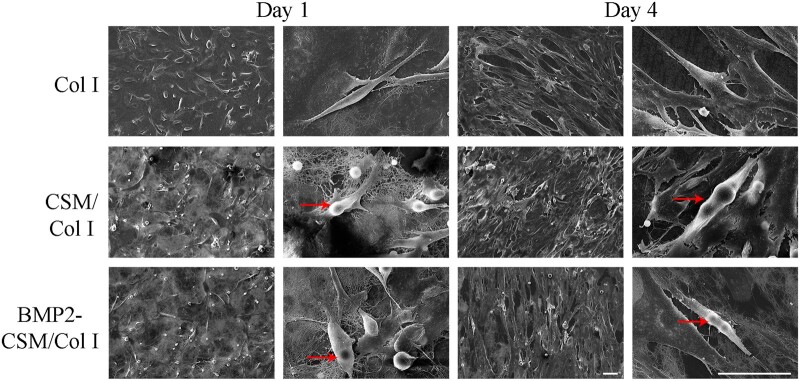
SEM Images of cell adhesion and proliferation at Days 1 and 4. Arrows indicate cell adhesion on microspheres. Scale bar = 50 μm

Cell adhesion was assessed using cytoskeleton staining and SEM. At Day 1, rice- or spindle-like USCs adhered and sparsely spread on the surface of hydrogels in 2D culture (Fig. 4C), and long-fusiform cells with good extension were observed in the interior of 3D hydrogels (Fig. 4D). More F-actin cytoskeletons were observed around the outer edge of cells, which meant many pseudopodia and extracellular matrix. At Day 4, the number of long-fusiform USCs increased greatly, and there were longer and denser F-actin microfilaments inside the cells. Interconnected cells attached, proliferated and extended with long and abundant pseudopodia both in 2D and 3D models (Fig. 4C and D). The results of SEM also showed cells adhered and spread on different hydrogels with good cellular morphology (Fig. 5).

### Osteogenic differentiation in 2D co-culture

**Figure 6.**
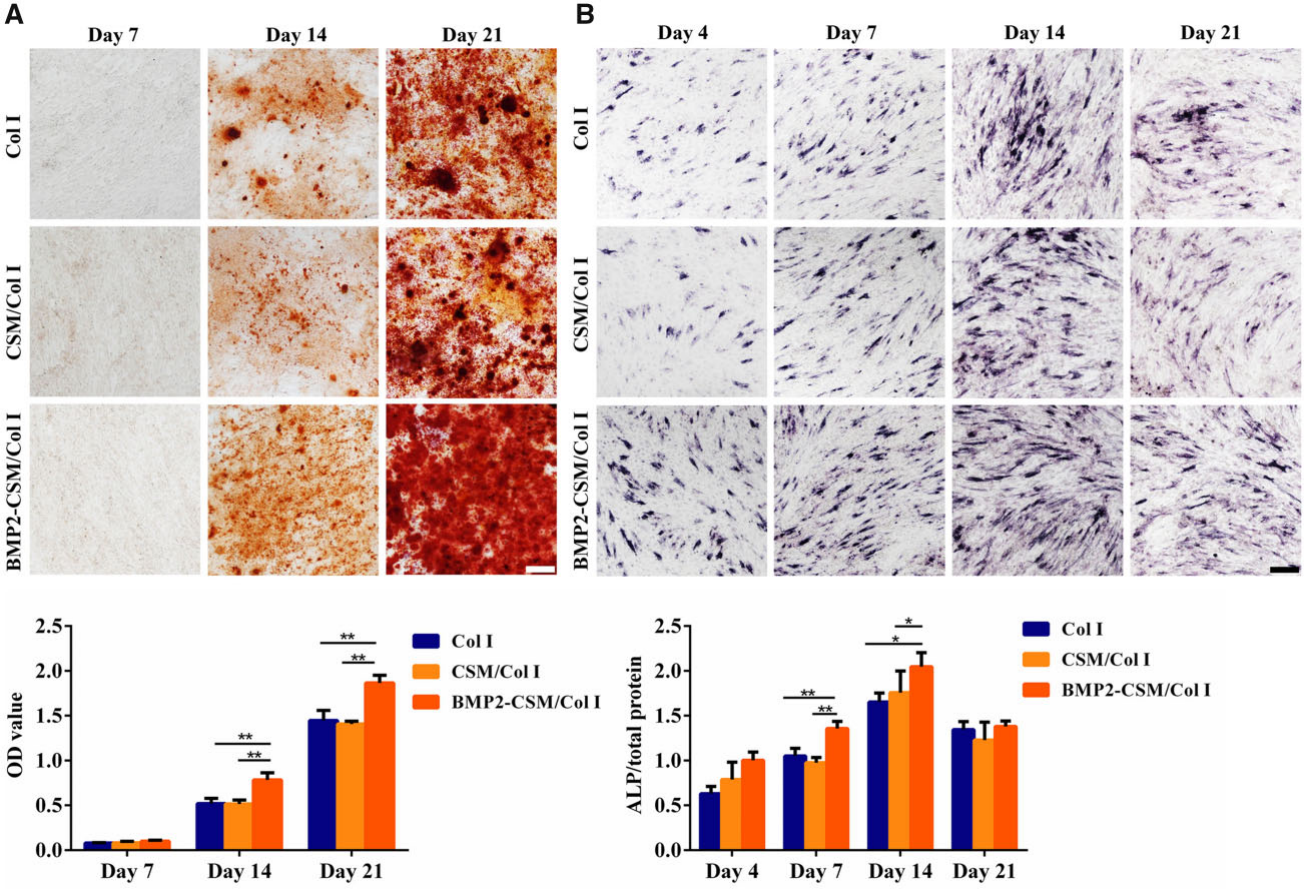
Osteogenic differentiation in 2D co-culture. (**A**) ARS staining and quantification at Days 7, 14 and 21. Scale bar = 100 μm. (**B**) ALP staining and quantification at Days 4, 7, 14 and 21. Scale bar = 100 μm. *P <* *0.05, **P <* *0.01

Mineral deposition was observed and quantified using ARS staining (Fig. 6A). No detectable calcium nodule was found after 7 days of osteogenesis induction. Calcium deposition increased obviously at Day 14, and then developed greatly at Day 21 in each group. Quantitative analysis of mineralization showed that the mineral level of BMP2-CSM/Col I group was statistically higher than others at Days 14 and 21 (*P *<* *0.05).

ALP expression was evaluated using ALP staining and quantitative assays (Fig. 6B). ALP expression was observed at Day 4, increased at Day 7, reached a peak at Day 14 and then decreased at Day 21. Quantitative results of ALP showed that ALP activity in the BMP2-CSM/Col I hydrogel group was significantly higher than that of other two groups at Days 7 and 14 (*P *<* *0.05).

### Osteogenic differentiation in 3D culture

**Figure 7.**
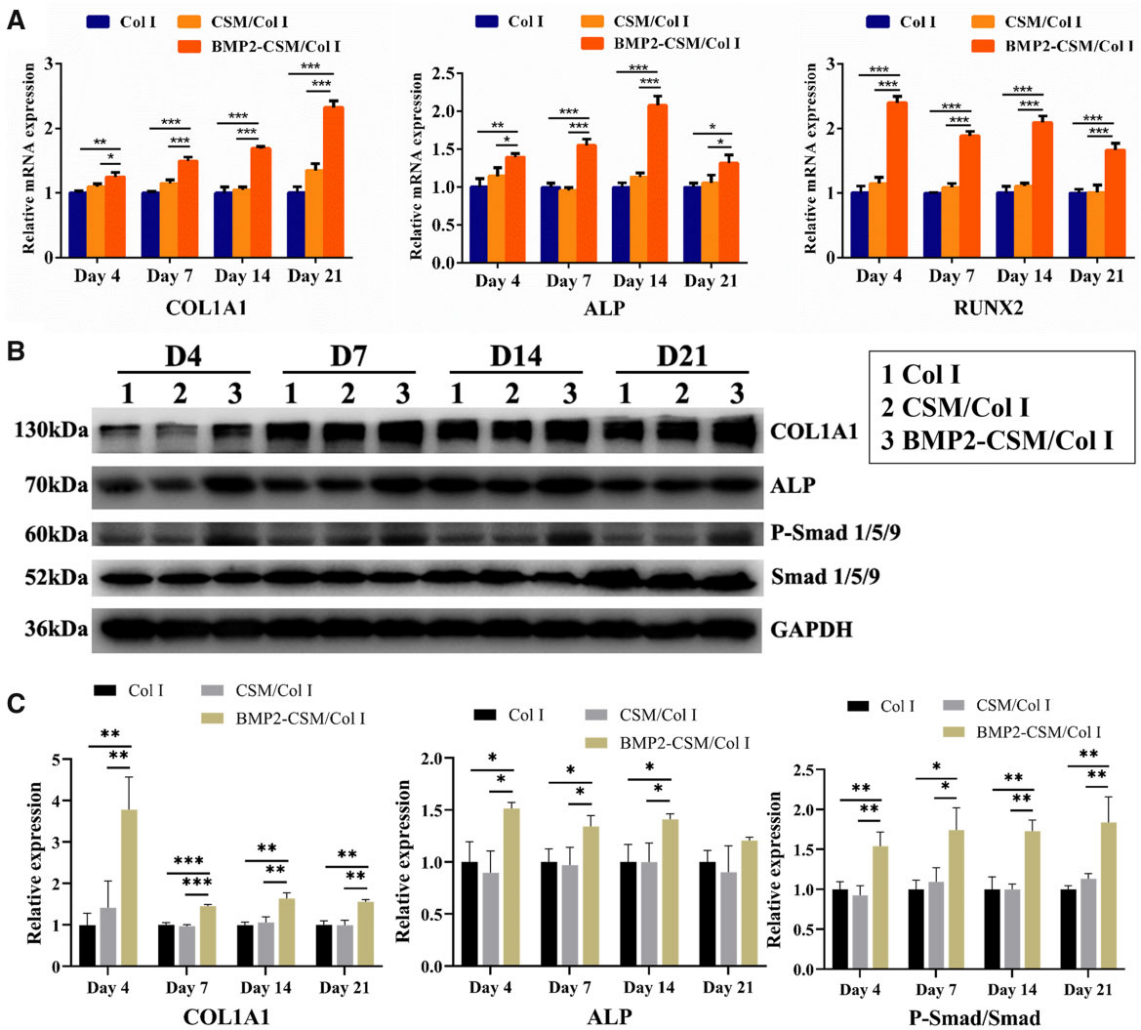
The expression levels of osteogenic genes and proteins. (**A**) The relative mRNA expression of COL1A1, ALP and RUNX2 at Days 4, 7, 14 and 21 after osteogenic induction. (**B**) Western blot analysis showed the protein expression levels of COL1A1, ALP, p-Smad 1/5/9 and Smad 1/5/9 at Days 4, 7, 14 and 21 after osteogenic induction. (**C**) Quantification analysis of protein expression. *P <* *0.05, **P <* *0.01, ***P <* *0.001

RT-PCR was used to measure the expression levels of osteogenic genes including COL1A1, ALP and RUNX2 (Fig. 7A). Gene expression levels in the BMP2-CSM/Col I hydrogel group were statistically up-regulated at Days 4, 7, 14 and 21 when compared with other groups. Simultaneously, no statistical difference of expression levels was detected between Col I hydrogel group and CSM/Col I hydrogel group.

Results of western blot analysis are shown in Fig. 7B and C. Compared with Col I hydrogel and CSM/Col I hydrogel groups, the protein expression of p-Smad 1/5/9 increased obviously in the BMP2-CSM/Col I hydrogel group. The protein expression of ALP was in accord with the results of ALP staining. The expression levels of COL1A1 and ALP in the BMP2-CSM/Col I hydrogel group were relatively higher than those of other groups.

### *In vitro* antibacterial properties

**Figure 8.**
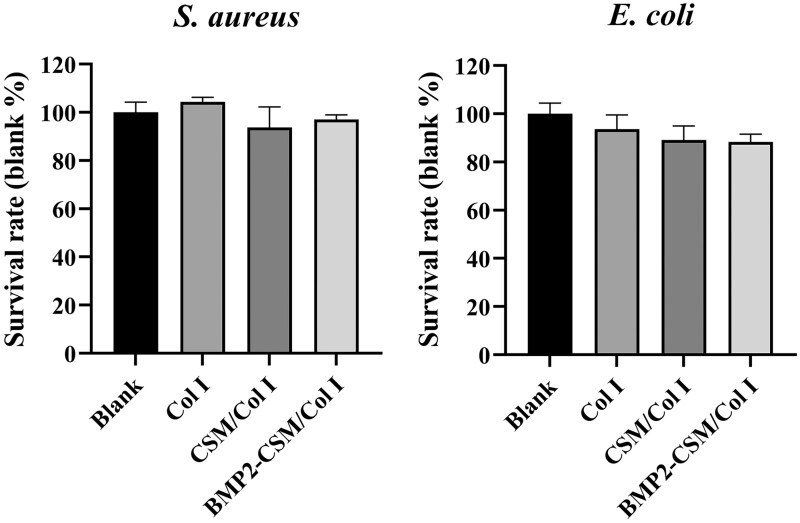
Evaluation of antibacterial capacity of hydrogels. *S.aureus and E.coli* survival rates at 12 h

Antibacterial properties are one of the important clinical conditions for stable healing and accelerated bone regeneration. Compared with the blank group, Col I, CSM/Col I and BMP2-CSM/Col I groups presented high survival rates both for *S.aureus* and *E.coli*, which suggested weak antibacterial effects of hydrogels (Fig. 8).

### *In vivo* studies

**Figure 9.**
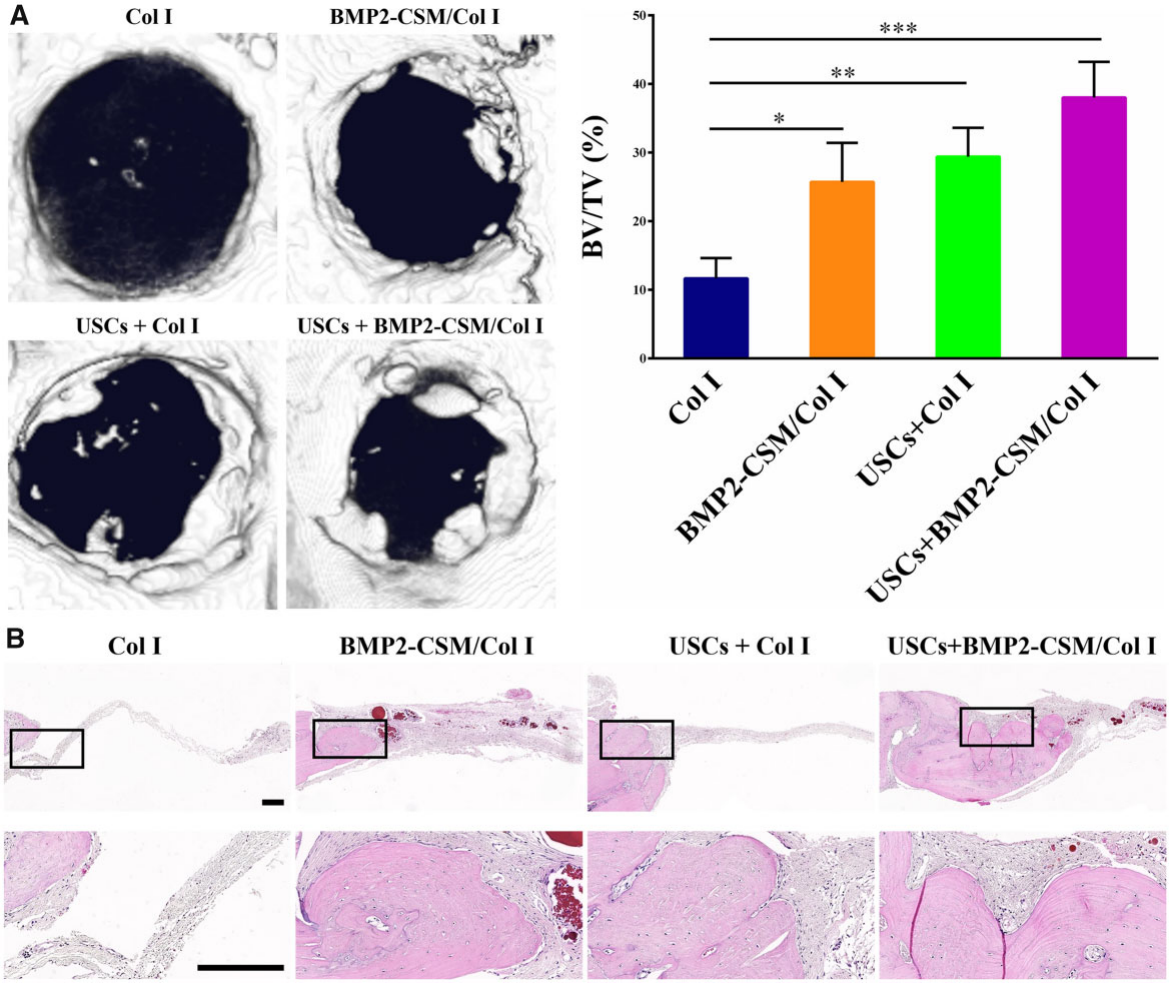
Results of *in vivo* experiments. (**A**) Representative micro-CT images and quantification using the ratio of new bone volume to total bone volume (BV/TV). *P <* *0.05, **P <* *0.01, ***P <* *0.001. (**B**) HE staining. The second row represents magnification images of the corresponding black rectangles in the upper row. Scale bar = 200 µm

Micro-CT was used to assess bone regeneration *in vivo* after 8 weeks of implantation (Fig. 9A). The BV/TV of the USCs + BMP2-CSM/Col I group was significantly higher than that of Col I, BMP2-CSM/Col I and USCs + Col I groups. BMP2-CSM/Col I and USCs + Col I groups exhibited more mineralization in the bone defects than Col I group, while there was no significant difference between BMP2-CSM/Col I and USCs + Col I groups.

The results of HE staining are shown in Fig. 9B. Compared with Col I, BMP2-CSM/Col I and USCs + Col I groups, more continuous collagen tissue and new bone were observed in the USCs + BMP2-CSM/Col I group, suggesting that there was more new bone regeneration. The volume of newly formed bone was similar in the BMP2-CSM/Col I group and the USCs + Col I group. In the Col I group, there were only some fibrous tissues in the bone defects. The results were consistent with the micro-CT images.

**Figure 10.**
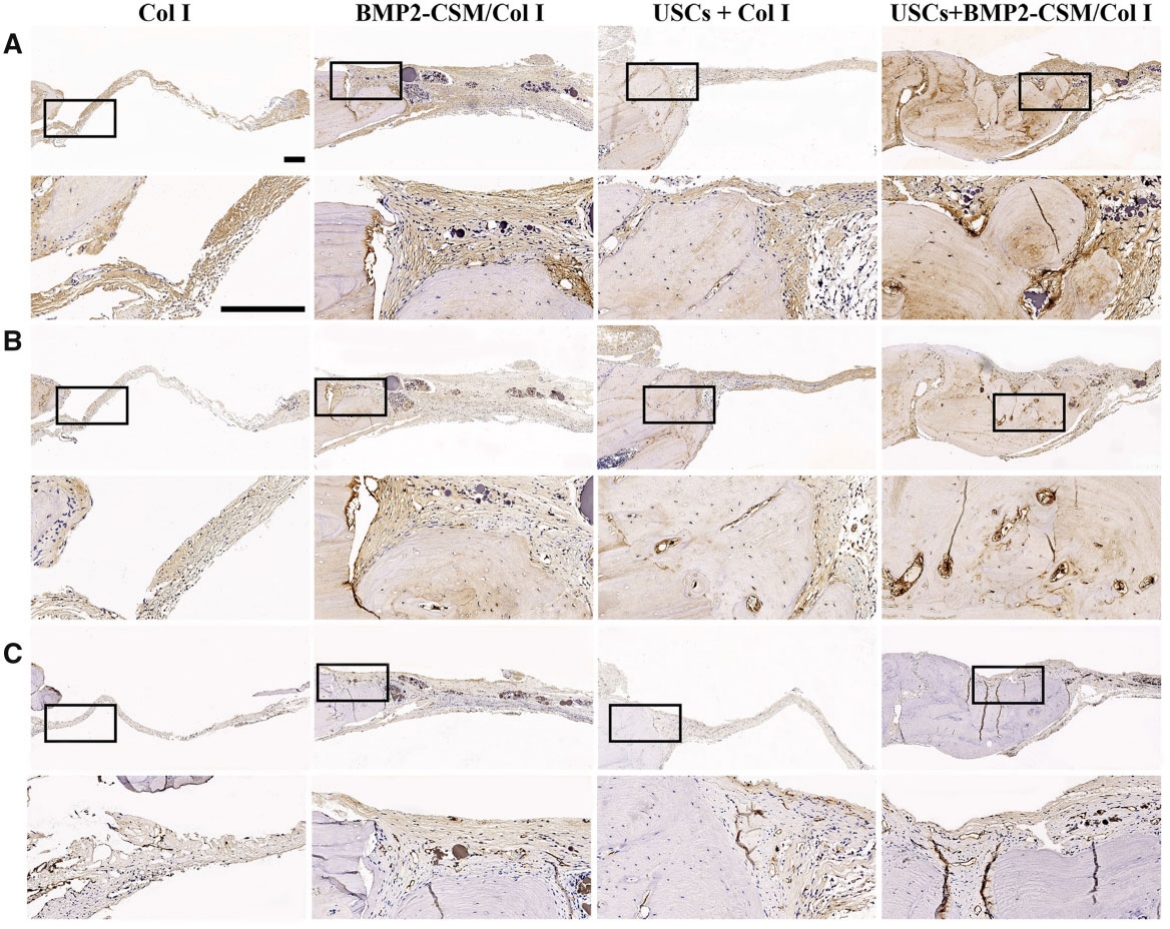
(**A**) COL1A1 immunohistochemical staining. (**B**) OCN immunohistochemical staining. (**C**) CD31 immunohistochemical staining. The second row represents magnification images of the corresponding black rectangles in the upper row. Scale bar = 200 µm

Representative images of immunohistochemical staining are shown in Fig. 10. For the evaluation of osteogenic potential, positive immunohistochemical staining of COL1A1 (Fig. 10A) and OCN (Fig. 10B) was observed, and there was more positive expression in the new bone tissue of the USCs + BMP2-CSM/Col I group. For the evaluation of angiogenic potential, there were more CD31-positive vessels in the USCs + BMP2-CSM/Col I group compared with other groups (Fig. 10C).

**Figure 11.**
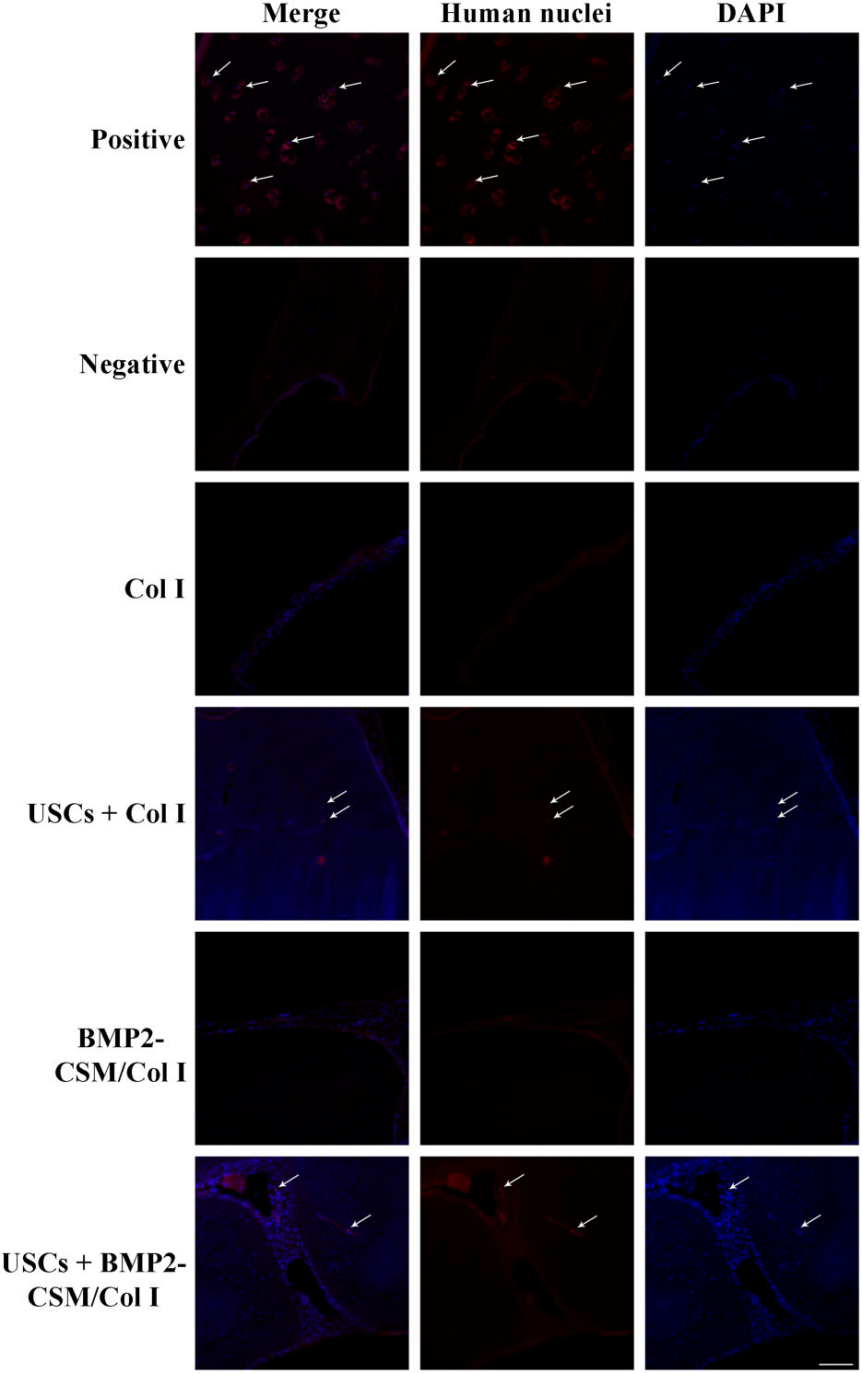
Implanted USCs were traced *in vivo* by immunofluorescent staining using anti-NuMA antibodies. Positive group: paraformaldehyde-fixed paraffin-embedded tissue sections of human femoral condylar cartilage. Negative group: paraformaldehyde-fixed paraffin-embedded tissue sections of SD rat cranial bone. Scale bar = 100 μm

Immunofluorescent staining was used to monitor the fate of USCs. NuMA-positive cells were observed in the new bone tissue of USCs + Col I and USCs + BMP2-CSM/Col I groups, while no positive expression was found in Col I and CSM/Col I groups (Fig. 11). The results indicated that USCs survived in the bone defects and enhanced the bone repair.

## Discussion

In this study, a BMP2-CSM/Col I hydrogel scaffold embedded with USCs was fabricated for bone tissue engineering. Under the influence of exogenously sustained-release BMP2 from BMP2-CSM/Col I hydrogels, the osteogenic differentiation of USCs was consistently enhanced at predetermined time intervals by activating BMP2/p-Smad 1/5/9 signaling pathways both in 2D and 3D modes *in vitro*. And BMP2-CSM/Col I hydrogels combined with USCs significantly promoted bone formation and neovascularization *in vivo* when compared with Col I, BMP2-CSM/Col I and USCs + Col I hydrogels.

Cell-based tissue engineering is a promising approach to facilitate bone regeneration [25]. MSCs have exhibited enormous potential for the application of bone tissue engineering. However, limited sources, invasive procedures or safety concerns restrict their clinical application, which urges the demand for more suitable cell sources [26, 27]. USCs can be non-invasively isolated from fresh urine, which could be as a renewable and abundant source of stem cells. USCs are multi-potential, and can differentiate into osteoblasts, chondrocytes, adipocytes, endotheliocytes and so on [10]. Besides, the application potential of USCs for bone healing has been demonstrated by several previous studies [12, 28].

BMP2 is capable of promoting bone formation in a dose–dependent manner within a reasonable range by enhancing the recruitment of osteoblast progenitor cells, angiogenesis and the osteogenic differentiation of MSCs. However, it has also been confirmed to be associated with side effects at high doses [16, 17]. Therefore, a sustained delivery system seems to be critical to the effectiveness of BMP2. As reported, CSMs have the ability to control the delivery of drugs to prolong their biological effects [29]. A sustained release of BMP2 from CSM can be more effective in bone healing when compared with a burst release alone. Meanwhile, Lee *et al*. [30] studied the release profile of BMP2, and found that BMP2 was released from Col I hydrogels for more than 28 days. In this study, the sustained-release delivery system of BMP2 was fabricated using Col I hydrogels combined with CSM, and the release profile suggested that this system could achieve sustained release of BMP2 for bone healing and avoid the side effects at high doses.

To evaluate the cytocompatibility of the scaffolds, cell proliferation, cell viability, cell adhesion and cell migration were assessed. All these results suggested that the composite scaffolds and pure Col I hydrogel scaffolds were biocompatible. USCs were able to adhere, survive and proliferate on the scaffolds. The proliferation slightly decreased at Day 10 in 2D culture, which might be due to the limited areas of the plate. Although previous studies have reported that BMP2 effectively accelerates the migration of a variety of cells, such as osteoblasts and bone marrow MSCs [31]. Few studies have explored stimulatory effects of BMP2 on USCs migration. In this study, a higher gap closure rate in a scratch assay was noted in the BMP2-CSM/Col I hydrogel group, which indicated that BMP2 released from the hydrogels was functional, and could accelerate the migration of USCs.

In this study, our results indicated that BMP2 increased the ALP activity and calcium content of USCs. ALP is the most widely recognized marker of osteoblast phenotypes [32]. The positive ALP and ARS staining indicated the osteogenic differentiation of USCs. Notedly, ALP typically expressed in the early stage of osteogenic differentiation [33], which may explain why the ALP expression decreased at Day 21. Besides, the ALP activity and calcium content were significantly higher in the BMP2-CSM/Col I hydrogel group suggested that exogenous BMP2 could stimulate the osteogenic differentiation and mineralization of USCs.

Furthermore, most of the osteogenic gene and protein expression significantly increased in the BMP2-CSM/Col I hydrogel group. RUNX2 is an important transcription factor for osteogenic differentiation [34]. RUNX2 can control the expression of bone-related genes, which are needed for osteogenic differentiation and bone formation [35]. The BMP2/Smad signaling pathway has been reported to promote the osteogenic differentiation of MSCs [36]. BMP2 induces the phosphorylation of Smad1 and Smad5, and then interacts with Smad4 to enter the nucleus to regulate osteogenic target genes [37]. All these results not only suggested USCs could be good seed cells for bone tissue engineering, but also indicated that BMP2 could induce and stimulate USCs to differentiate into the osteoblasts through the BMP2/Smad signaling pathway.

Results of micro-CT and histologic staining revealed that USCs-seeded BMP2-CSM/Col I hydrogel scaffolds had the best bone formation ability, suggesting that cells played an important role in bone regeneration. Similar results were revealed by Bruder *et al.* [38], who reported that bony union was achieved in dog femoral defects after implantation with MSCs-seeded scaffolds compared with scaffolds alone. Immunohistochemical staining showed that more positive staining of COL1A1 and OCN was observed in the USCs + BMP2-CSM/Col I group, indicating that the scaffold was able to stimulate the repair of bone defects, which is consistent with the *in vitro* data.

Angiogenic capability is critical for bone tissue engineering [39]. Immunohistochemical staining showed that more CD31-positive blood vessels were observed in the USCs + BMP2-CSM/Col I group compared with other groups, suggesting that the combination of USCs and BMP2-CSM/Col I hydrogels could improve bone regeneration effects by promoting angiogenesis. Guan *et al.* [28] have found that USCs could secrete a large amount of VEGF proteins. In addition, BMP2 was reported to stimulate angiogenesis by increasing the secretion of VEGF from osteoblasts [40]. We speculated that the secretion of VEGF from USCs and osteoblasts may induce the ingrowth of blood vessels into scaffolds.

Stem cell transplantation alone can help achieve good outcomes under certain circumstance, but an ideal delivery system is still needed to improve survival rate, induce phenotypic changes and promote efficient differentiation. Some studies have demonstrated the potential of the combination of USCs, growth factors and other bioactive materials in tissue regeneration. Liu *et al.* [41] fabricated an alginate microspheres/Col I sustained-release system embedded with USCs and multiple growth factors (VEGF, IGF-1, FGF-1, PDGF, HGF and NGF), and demonstrated that the combination of growth factors and USCs improved the survival of grafted cells, enhanced the revascularization and innervation, promoted the myogenic differentiation, and even recruited more resident cells to the implanted site. Guan *et al.* [28] showed calcium silicate (CS) ion extracts stimulated the proliferation and osteogenic differentiation of USCs by activating the Wnt/β-catenin signaling pathway *in vitro*, and PLGA/CS scaffolds enhanced the osteogenic differentiation of USCs and vascularization *in vivo*. In this study, we demonstrated BMP2-CSM/Col I hydrogels enhanced the osteogenic differentiation of USCs *in vitro*, and USCs survived in the bone defects and enhanced the bone repair *in vivo*. The combination of BMP2-CSM/Col I hydrogels and USCs promoted the bone regeneration, which provided a novel strategy for the repair of bone defects.

In addition, USCs are of great interest in tissue regeneration due to immunomodulation and paracrine effects. Some studies demonstrated that USCs participated in some immune response by regulating the phenotype and function of immune cells [42–44]. Zhou *et al.* revealed that grafted USCs down-regulated Th1/Th17 immune responses and reduced inflammation in an inflammatory bowel diseases rodent model [43]. The paracrine effect of stem cells is of vital importance in tissue repair. Stem cells can modulate the micro-environment and recruit resident cells by secreting many paracrine factors. Similarly, USCs enhanced the proliferation and migration of vascular endothelial cells by the paracrine action [45, 46].

## Conclusions

USCs, a promising cell source in bone tissue engineering, possessed the capacity of stable proliferation and multi-potential differentiation. The sustained release of BMP2 promoted the differentiation of USCs into osteoblasts through the BMP2/Smad signaling pathway *in vitro*. BMP2-CSM/Col I hydrogels embedded with USCs significantly enhanced new bone formation *in vivo*. Taken together, the combination of USCs and BMP2-CSM/Col I hydrogels have a great application potential for bone tissue engineering.
